# Anti-amphiphysin encephalitis: Expanding the clinical spectrum

**DOI:** 10.3389/fimmu.2023.1084883

**Published:** 2023-04-05

**Authors:** Yueqian Sun, Xiaoxiao Qin, Danxia Huang, Ziqi Zhou, Yudi Zhang, Qun Wang

**Affiliations:** ^1^ Department of Neurology, Beijing Tiantan Hospital, Capital Medical University, Beijing, China; ^2^ Department of Neurology, Fujian Medical University Affiliated First Quanzhou hospital, Quanzhou, China; ^3^ National Center for Clinical Medicine of Neurological Diseases, Beijing, China; ^4^ Beijing Institute of Brain Disorders, Collaborative Innovation Center for Brain Disorders, Capital Medical University, Beijing, China

**Keywords:** amphiphysin, autoimmune encephalitis, clinical features, treatment, prognosis

## Abstract

**Objective:**

An analysis of the clinical features of autoimmune encephalitis accompanied by anti-amphiphysin antibodies.

**Methods:**

The data of encephalitis patients with anti-amphiphysin antibodies were retrospectively evaluated, including demographics, neurological and laboratory findings, imaging, treatment, and prognostic predictions.

**Results:**

Ten patients aged between 29 and 78 years (median age 52 years) were included. The male: female ratio was 4:6. Limbic encephalitis was found in nine patients while epileptic seizures were present in seven patients. All patients showed anti-amphiphysin antibody positivity in sera while one ninth was positive for CSF antibody. The EEG findings were abnormal, including reductions in background activity, and the presence of diffuse slow waves, sharp waves, and spikes and waves. Five patients showed signs of increased T2 signals in the medial temporal lobe on MRI while PET showed either hyper- or hypo-metabolic changes in several brain regions, including the temporal lobe, hippocampus, basal ganglia, frontal and parietal cortices. Nine of ten patients were treated with immunotherapy, with improvements of varying degrees. There was a significant reduction in seizure frequency, and all patients were seizure-free at last follow-up.

**Conclusion:**

Autoimmune encephalitis with anti-amphiphysin antibodies has a variety of clinical manifestations. The most common symptom is limbic encephalitis. Although relief from seizures can be achieved relatively easily, many patients suffer psychiatric, cognitive, and sleep sequelae. The disease was found to be associated with a lower incidence of cancer than has been previously reported for paraneoplastic neurologic syndromes.

## Introduction

Autoimmune encephalitis (AE) is a form of encephalitis resulting from autoimmune reactions (1). It is generally acute or subacute and has an annual incidence of approximately 0.8/100,000 (2). The typical presentation includes behavioral disorders, psychiatric symptoms, cognitive impairment, seizures, and impaired consciousness (1, 3). Since the first description of AE, characterized as anti-*N*-methyl-d-aspartate receptor (NMDAR) antibodies in 2007 (4), a variety of encephalitis-associated autoantibodies directed against components of the neuron have been discovered. These include antibodies against leucine-rich glioma-inactivated 1 protein (LGI1), contactin-associated protein-like 2 (CASPR2), γ-aminobutyric acid B receptor (GABA_B_R), α-amino-3-hydroxy-5-methyl-4-isoxazole-propionic acid receptor (AMPAR), glycine receptor (GlyR), and so on.

Amphiphysin is a Bin/Amphiphysin/Rvs (BAR) domain containing protein that is involved in clathrin-dependent endocytosis during inhibitory neurotransmission (5). Its SH3 domain acts as a binding site during the formation of clathrin-coated intermediates (6). Amphiphysin has been associated with paraneoplastic neurological syndromes (PNS) in breast or small-cell lung cancers (7). The presence of anti-amphiphysin autoantibodies is most commonly associated with limbic encephalitis (LE) and stiff-person syndrome (8–10). Apart from the above, a number of case reports and small case series have reported a link between anti-amphiphysin antibodies and brainstem encephalitis (11), myelopathy (12, 13), peripheral neuropathy (11, 14), and cerebellar dysfunction (11). Anti-amphiphysin encephalitis is usually treated in the same way as other AE. First-line therapies are corticosteroids, intravenous gamma globulin (IVIG), and plasmapheresis (PLEX) and second-line treatment involves the addition of rituximab or cyclophosphamide in patients unresponsive to first-line therapy (15). Immunotherapy is effective for treating encephalitis patients with anti-amphiphysin antibodies, especially those without tumors (15). Treatment of tumors should be performed without delay to improve outcomes (1).

However, an in-depth understanding of amphiphysin-IgG-associated encephalitis is lacking. Here, we describe the clinical features of 10 anti-amphiphysin encephalitis patients and their outcomes after immunotherapy. A literature review of amphiphysin-antibody-associated neuropathy was also performed.

## Materials and methods

Patients with anti-amphiphysin antibody positivity in serum or cerebrospinal fluid (CSF) admitted to the Beijing Tiantan Hospital between 2018 and 2022 were reviewed. Ten patients fitting the criteria of autoimmune encephalitis updated in 2016 were retrospectively enrolled (1).

### Laboratory tests

Antibody testing of the sera and/or CSF of all patients with suspected autoimmune encephalitis was performed. The spectrum of antibodies tested included all known neuronal autoantibodies (NMDAR, LGI1, CASPR2, GABABR, AMPAR, and glutamic acid decarboxylase 65 [GAD65]) and classical paraneoplastic antibodies (Hu, Yo, Ri, Ma2, CV2, and amphiphysin). Samples were analyzed semi-quantitatively by cell-based assays (Euroimmun, Lübeck, Germany) and immunohistochemistry was performed by the Neuroimmunology Laboratory of the Peking Union Medical College Hospital (Beijing, China).

### EEG and imaging

Routine long-term video-electroencephalography (EEG) recording was performed with standard 10-20 system electrodes. Magnetic resonance imaging (MRI) was performed on each patient with a 3T MRI system (Signa HD xt *3T* Volume, *GE*, *GE* Healthcare, USA) while ^18^F-FDG positron emission tomography (PET) scans were done using a PET/CT scanner in all except two patients due to concerns of radiation exposure (Elite Discovery, GE HealthCare, Fairfield, CT, USA).

### Functional assessment and evaluation of outcomes

For most patients (except Patients #4, #9, #10), the Montreal Cognitive Assessment (MOCA) and the Mini-Mental State Examination (MMSE) were used to evaluate cognition. The modified Rankin Scale (mRS) was used for the assessment of clinical outcomes at discharge and follow-up.

### Literature review

A comprehensive literature search for articles published between January 2002 and October 2022 was conducted in the PubMed database using the search terms “Amphiphysin” and “encephalitis”. All results of the literature search were reviewed and relevant information was extracted and summarized. This search yielded 42 publications, from which 13 papers were included in the final review.

## Results

### Clinical characteristics

The ratio of males to females was 4:6 and the median onset age was 52 years old (range 29-78 years old). Most patients did not experience prodromal symptoms, with only one having fever and headache (Patient #9). The details of the clinical presentations are summarized in **Table 1**. Additionally, Patient #1 had a history of invasive ductal carcinoma in the right breast that was completely resected before the onset of encephalitis.

**Table 1:** Clinical manifestation of anti-amphiphysin encephalitis.

Patient	Gender	Age	LE			Stiffness	Pain	Limb weakness/numbness	Paresthesia	Ataxia	Sleep disorder	Dysautonomia
			Seizure	Cognitive disorder	Mental and behavior disorder							
1	F	42		+ (MMSE=28/MOCA=25)		+	+	+	+		+ (DIS)	+ (hidrosis)
2^a^	M	54		+ (MMSE=22/MOCA=13)	+ (apathy)							
3	M	69	+	+ (MMSE=29/MOCA=24)							+ (RBD)	
4	M	77	+	+	+ (irritable)					+		
5	F	42	+	+ (MMSE=30/MOCA=20)								
6	M	59	+	(MMSE=29/MOCA=29)								
7	F	42	+	+ (MMSE=26/MOCA=21)	+ (anxiety, depression)			+	+	+		
8	F	78	+	+ (MMSE=27)							+ (DMS)	
9	F	29					+	+		+		
10	F	50	+	+								

DIS, difficulty initiating sleep; DMS, difficulty maintaining sleep; LE, limbic encephalitis; MMSE, Mini-Mental State Examination; MoCA, Montreal Cognitive Assessment; RBD, rapid-eye-movement sleep behavior disorder. a, anti-AMPAR and anti-CV2 antibodies were positive in serum. ; +, positive.

In this study, limbic encephalitis was the most common symptom. Seven patients suffered from epileptic seizures, with four having more than one type of seizure. Four patients experienced focal and bilateral tonic-clonic seizures, preceded by focal impaired awareness. One patient had myoclonic seizures involving both arms (Patient #1). Three of the seven patients suffered status epilepticus (Patients #4, #7, and #10). One patient was admitted to the Intensive Care Unit (ICU) due to hypoxemia during a seizure attack (Patient #10). In addition, 8 patients had cognitive disorder, of which 6 mentioned mild memory loss. Mental and behavioral disorders were infrequently observed, with only three patients reporting apathy, irritability, and anxiety-depression, respectively. In addition, sleep disorders were reported by patients #1, #3, and #8, which presented as difficulty initiating sleep (DIS), rapid-eye-movement sleep behavior disorder (RBD), and difficulty maintaining sleep (DMS).

None of the patients showed rigidity or spasm involving the axial muscles, described as stiff-person syndrome (16). Only one (Patient #1) showed episodic left upper limb extension with wrist adducent stiffness, induced by massage and lasting 1-2 minutes each time, always accompanied by sweating. Weakness and pain were present when lifting the upper right limb during the attack.

Sensory neuronopathy was found in two patients. In one patient (Patient #1), pruritus was easily induced by touching, usually on the upper limbs, shoulder, and back. Another patient (Patient #7) experienced soreness, numbness, and hyperalgesia in the right limbs. Furthermore, one (Patient #9) experienced numbness of the extremities, which was later confirmed by spinal cord MRI to be myelitis. She also showed impaired vision in the right eye and the visual acuities were 20/25 OD and 20/20 OS.

Cerebellar ataxia occurred in three patients (Patients #4, #7, and #9). All showed unstable walking, with one showing right limbs ataxia on physical examination, while no dysarthria or postural ataxia was observed.

### Auxiliary examinations

Nine patients had both serum and CSF samples tested, with one (Patient #8) not undergoing CSF testing due to rejection of the lumbar puncture. All showed anti-amphiphysin antibody positivity in the sera while positivity was only found in the CSF in one patient. Anti-AMPAR and anti-CV2 antibodies were also present in the serum of Patient #2. Detailed results of the laboratory tests are shown in **Table 2**.

**Table 2:** Laboratory and neuroimaging data of anti-amphiphysin encephalitis.

Patient	CSF			Tumor marker	MRI	EEG	PET-CT	
	WBC (n/μL)	Protein (mg/dL)	SOB				Brain/spinal	Whole body
1	4	50.2↑	+	Normal	Increased T2 signal in right MTL	NA	Right MTL hypermetabolism	Normal
2	8	26.64	+	NSE 65.5 ng/mL, Cyfra21-1 6.9 ng/mL, PROGrp 4297 pg/mL	Normal	NA	Right PL, bilateral FL hypometabolism, bilateral BG hypermetabolism	Lung and mediastinal mass with hypermetabolism
3	4	49.42↑	+	CA19-9 27.22 U/mL, NSE 20.72 ng/mL, TPSA 4.31 ng/mL	Increased T2 signal in left MTL	Sharp waves in left MTL	Left MTL hypometabolism	Normal
4	6	46.7↑	+	CA19-9 3.64 ng/mL, TPSA 6.52 ng/mL	DWI showed diffusion restriction in bilateral thalamus with blurred structure	NA	Bilateral central cortex, left fronto-parietal para-sagittal, BG, right thalamus hypermetabolism	Normal
5	1	17.26	–	AFP 11.2 ng/mL	Decreased volume of left hippocampus	Sharp waves in right MTL	Right MTL hypometabolism	Normal
6	34	22.85	+	CA72-4 11.97 U/mL	Increased T2 signal in the left MTL	Normal	Left MTL hypometabolism	Normal
7	2	17.62	+	NSE 21.2 ng/mL	Normal	Continuous sharp waves in the left central-parietal area	Left MTL hypermetabolism	NA
8	NA	NA	NA	Normal	Normal	NA	NA	NA
9	40	92.24↑	+	CA19-9 28.42 U/mL	Increased T2 signal and enhancement in posterior horn of the spinal cord (C2-7)	NA	Spinal cord hypermetabolism	Normal
10	9	58.3↑	NA	Normal	Increased T2 signal in bilateral MTL	Normal	NA	NA

AFP, alpha-fetoprotein; BG, basal ganglia; CA, carbohydrate antigen; CSF, cerebrospinal fluid; Cyfra21-1, cytokeratin 19 fragment antigen2 -1;EEG, electroencephalogram; FL, frontal lobe; MTL, medial temporal lobe; MRI, magnetic resonance imaging; NA, not applicable; NSE, neuron-specific enolase; PET-CT, positron emission tomography-computed tomography; PL, parietal lobe; PROGrp, Progastrin-releasing peptide; TPSA, total prostate specific antigen; SOB, CSF-specific oligoclonal bands; WBC, white blood cell; +, positive; -, negative.

The CSF results showed evidence of central nervous system (CNS) inflammation and abnormalities of the immune system. Two of nine patients had elevated white blood cell (WBC) counts (>10 n/μL) while five of nine patients had protein concentrations exceeding 45 mg/dL; the ranges of the WBC counts and protein levels were 1-40 n/μl and 17.26-92.24 mg/dL, respectively. CSF-specific oligoclonal bands were detected in most patients (7/8) and eight of the 10 patients showed abnormal levels of tumor markers (**Table 2**). One of these patients showed highly elevated PROGrp (4297 pg/mL, normal range 0-65.7 pg/mL), indicating a high likelihood of lung cancer, which was confirmed to be small-cell lung cancer by subsequent PET-CT.

**Figure 1:**
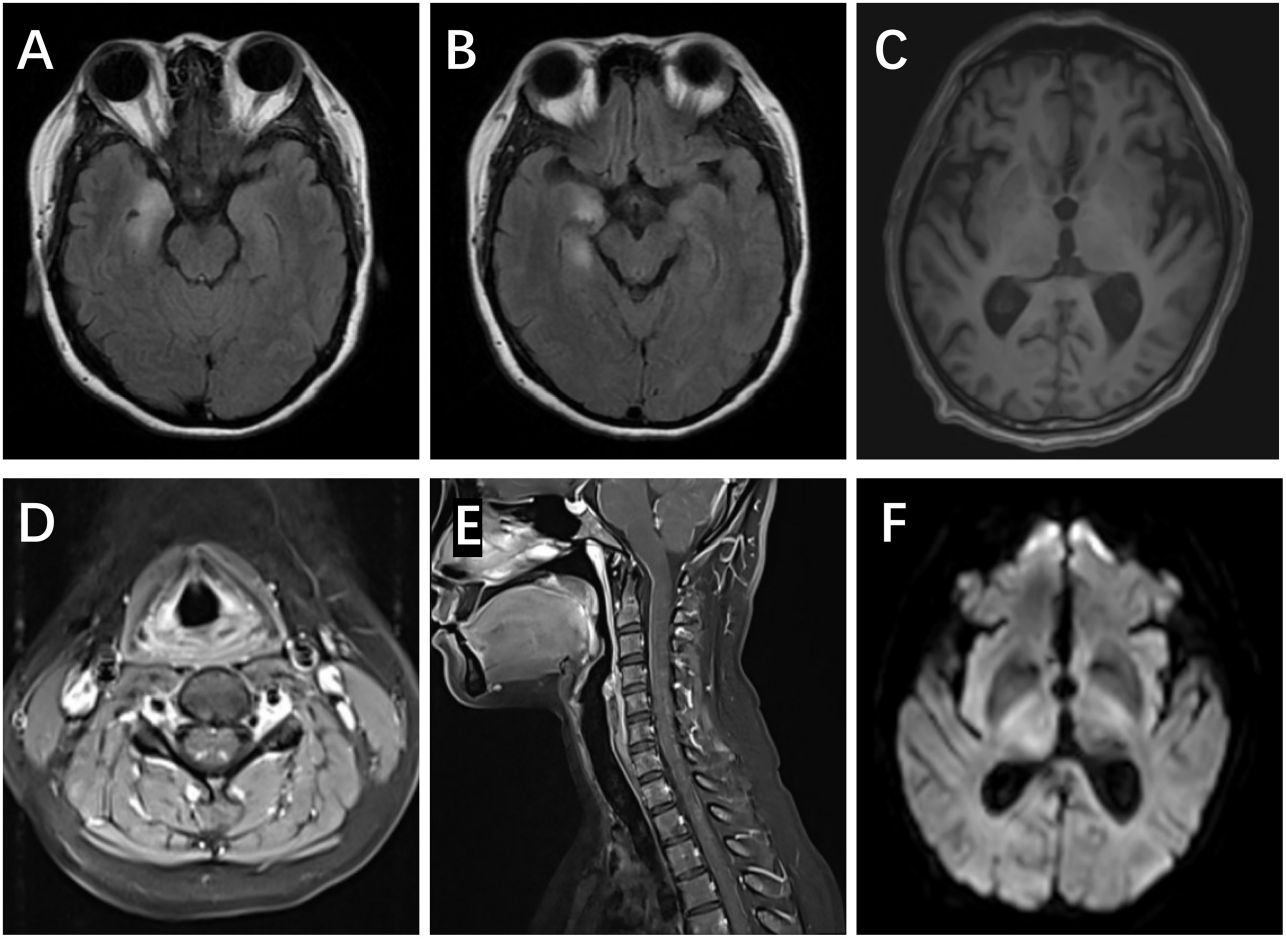
MRI scans in anti-amphiphysin encephalitis. **(A, B)** Right FLAIR hyperintensities in the medial temporal lobes, including both the amygdala and hippocampus (Patient #1). **(C)** The blurred structure of the bilateral thalamus (Patient #4). **(D, E)** Increased T2 signal and enhancement in posterior horn of the spinal cord (C2-7) (Patient #9). **(F)** Restriction of DWI in the right thalamus (Patient #4).

**Figure 2:**
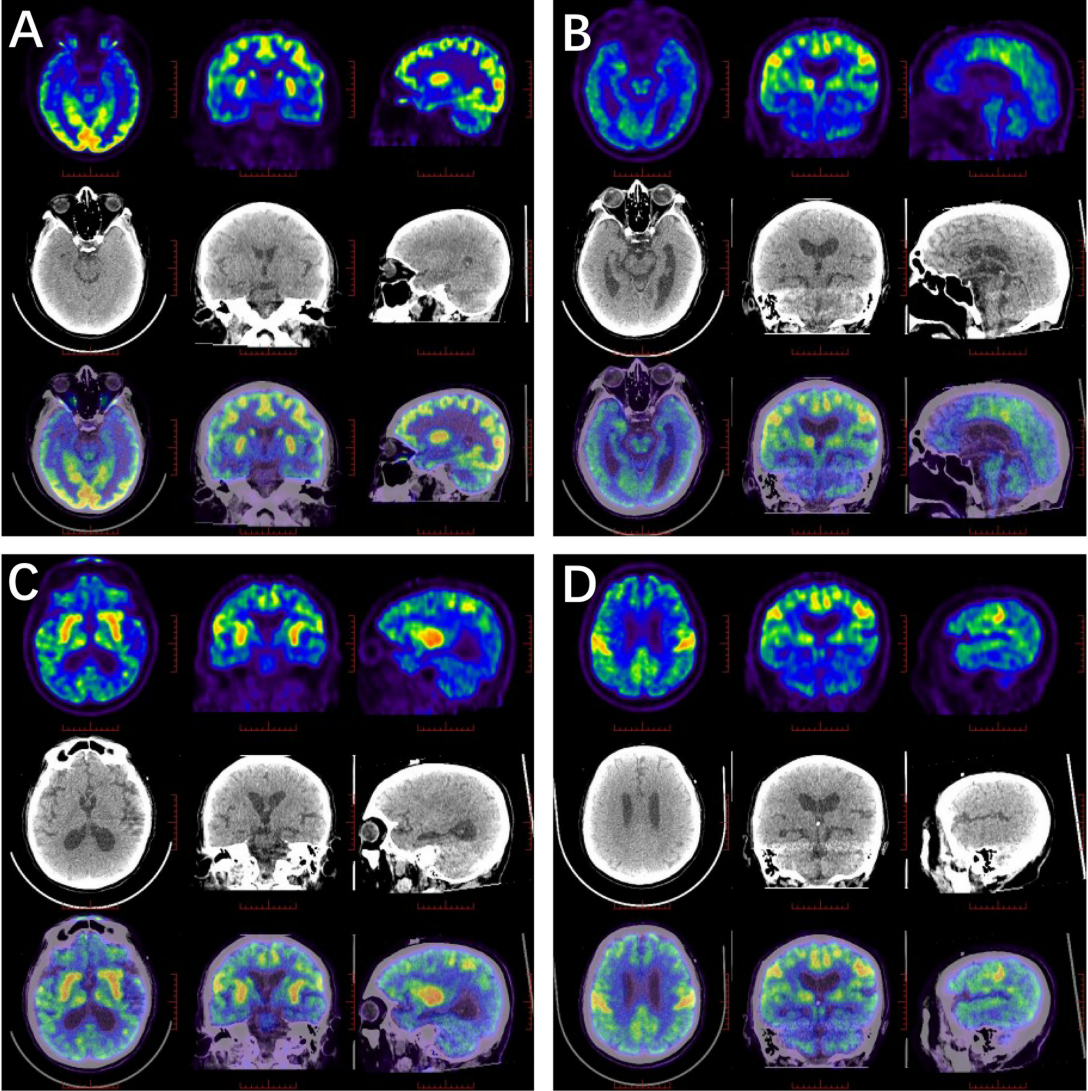
^18^F-FDG PET/CT scan in anti-amphiphysin encephalitis. **(A)** Glucose metabolism decreased in the right hippocampus (Patient #1). **(B–D)** High FDG uptake in the bilateral central cortex, left fronto-parietal para-sagittal, bilateral caudate heads, putamen and right thalamus, while the remaining brain cortex was diffusely and slightly decreased (Patient #4).

**Figure 3:**
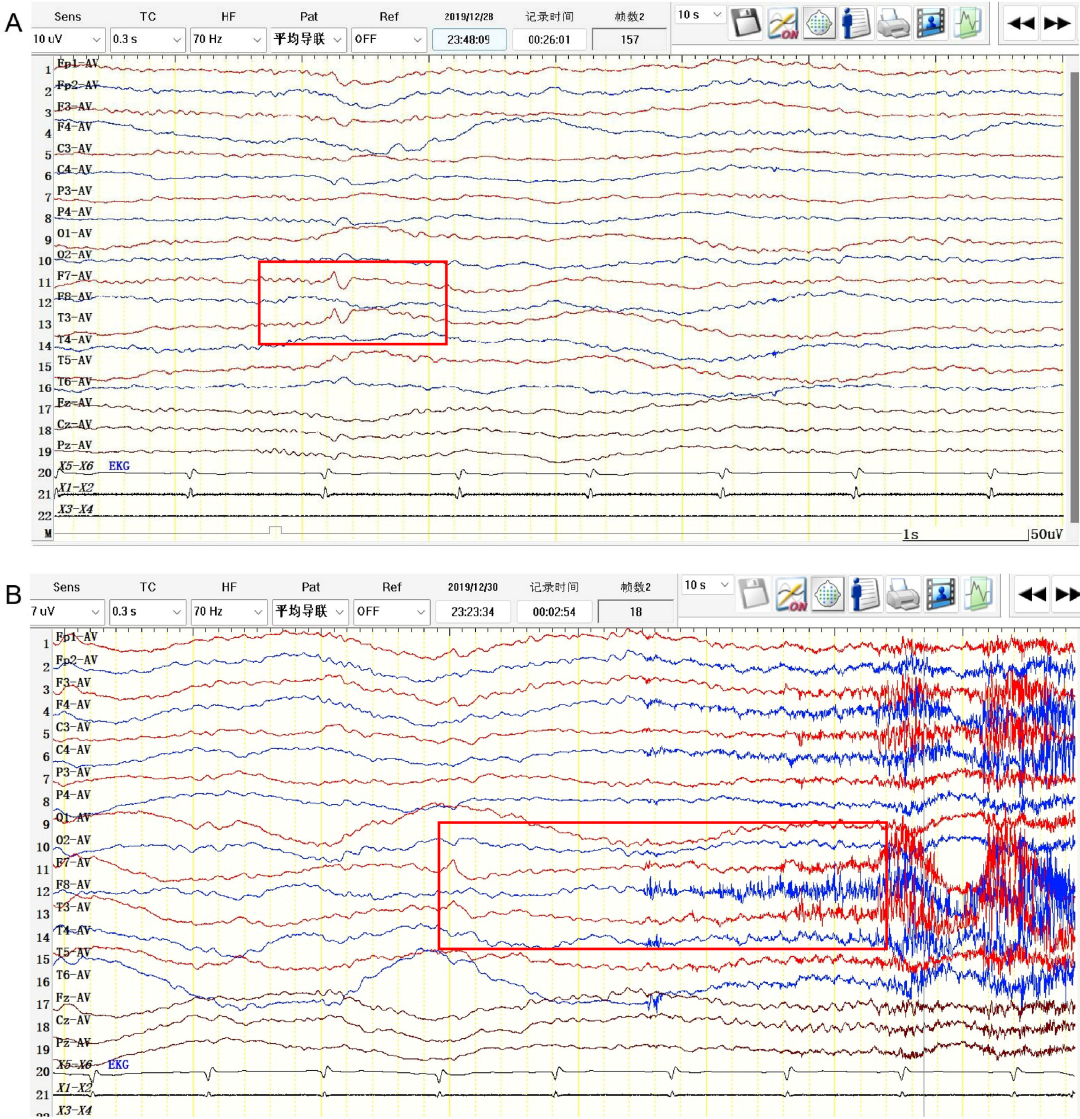
EEG pattern in anti-amphiphysin encephalitis. (Patient #3). **(A)** Interictal EEG showed sharp and waves in the left anterior temporal region (F7, T3). **(B)** Ictal EEG showed low-amplitude fast rhythmic activity with evolution originating from left temporal region.

Five patients showed involvement of the hippocampus on MRI (four unilateral, one bilateral). In Patient #4, to our surprise, the structure of the bilateral thalamus was blurred, with T2 high-signal lesions in the right thalamus (**Figure 1**). In addition, evidence of non-specific lacunar infarctions and white-matter lesions were common in our older patients, although these were not AE-related. Brain PET-CT revealed either hyper- or hypo-metabolic changes in several brain regions including the temporal lobe, hippocampus, basal ganglia, and the frontal and parietal cortices (**Figure 2**). EEG was performed on a total of five patients, with abnormal findings in 3/5 (60%), including diffuse slow waves and sharp waves (**Figure 3**).

Furthermore, Holter electrocardiogram (ECG) examinations revealed asymptomatic bradycardia in one patient (Patient #6). Two patients received breast ultrasound examinations and were found to have benign hyperplastic nodules (Patients #7 and #9).

### Treatment and prognosis

Nine patients were given first-line treatment, while one refused immunotherapy due to concern about side effects (Patient #8). Of the treated patients, five received IVIG administration only, three were treated with IVIG combined with steroids, and one was treated only with corticosteroids. In addition, patients received anti-seizure medications (ASMs), including sodium valproate (VPA), oxcarbazepine (OXC), levetiracetam (LEV), lacosamide (LCM), and lamotrigine (LTG).

All patients were followed up for at least three months after discharge, and the sequelae are summarized in **Table 3**. Patients #5, #7, #9, and #10 undertook sequential oral corticosteroids after discharge. None of the ten patients undertook second cycle of IVIG or corticosteroids treatment. A comparison of the mRS scores before and after treatment indicated the effectiveness of treatment, as the scores of 7 patients had decreased by at least one point. The seizure frequency was significantly reduced after treatment and all patients retrieved seizure free at last follow-up. However, sleep disorders, cognitive impairment and emotional disorders persisted on follow-up.

**Table 3:** Treatment and prognosis of anti-amphiphysin encephalitis.

Patient	Treatment		Prognosis		
	Immunotherapy	ASMs	Symptoms left at last follow-up	mRS at discharge	mRS in 3 months
1	IVIG; chemotherapy	(-)	Right upper limb numbness, painful, and pruritus; lower limbs weakness; sleep disorder (15 m)	4	4
2	IVIG; chemotherapy	(-)	Hallucination, irritable, hyperhidrosis, urinary retention, and constipation (14 m)	3	1
3	IVIG	OXC	Sleep disorder, anxiety-depression (33 m)	1	1
4	IVIG	LEV	Numbness of extremities, walking instable, anxiety-depression (10 m)	3	2
5	Corticosteroids	LCM	Anxiety-depression, anorexic; sleep disorder (restless sleep) (3 m)	2	1
6	IVIG	LEV	Memory loss (4 m)	2	1
7	IVIG + corticosteroids	OXC, LEV, LTG	Weakness of right limbs, walking instable (5 m)	3	2
8	(-)	LCM	Memory loss, sleep disorder (3 m)	1	1
9	IVIG + corticosteroids	(-)	Headache, back numbness (10 m)	2	1
10	IVIG + corticosteroids	LEV, VPA	Memory loss (17 m)	4	1

ASMs, anti-seizure medications; IVIG, intravenous immune globulin; LCM, lacosamide; LEV, levetiracetam; LTG, lamotrigine; m, month; mRS, modified Rankin Scale; OXC, oxcarbazepine; RBD, rapid-eye-movement sleep behavior disorder; VPA, valproic acid; (-), none; +, plus.

## Discussion

We retrospectively enrolled 10 patients diagnosed with amphiphysin-antibody-related encephalitis. The male: female ratio was 4:6, which is consistent with previous studies showing a greater frequency among female patients (17–19). The age of onset was during middle or old age, consistent with previously published data (17–19).

Anti-amphiphysin encephalitis has a variety of clinical manifestations, including limbic encephalitis, stiffness, limb weakness/numbness, paresthesia, ataxia, sleep disorders, and dysautonomia, amongst others. Of these, limbic encephalitis is the most common symptom. Notably, patients who conform to the AE criteria should be suspected of having anti-amphiphysin antibodies when presenting with stiffness or paresthesia, even though it is rare.

Stiff-person syndrome (SPS) is a characteristic manifestation in amphiphysin-antibody-positive cases, although it did not appear as frequently as expected in our cohort (18–20). Amphiphysin-antibody-related SPS involves neck and arm instead of axial muscles of trunk and thus usually confused clinicians (18). One patient in our study presented unilateral upper limb stiffness, accompanied by paresthesia and dysautonomia. She fitted the diagnosis of progressive encephalomyelitis with rigidity and myoclonus (PERM), a severe immunophenotype of stiff-person spectrum disorder (SPSD) (21). This patient showed strong anti-amphiphysin antibody positivity in her serum, much higher than that seen in the other patients, suggesting a potential link between higher antibody titers and SPS severity. However, the relationship between antibody titers and the severity of clinical manifestation is still not clear in AE and requires future in-depth research.

Patients also demonstrated symptoms of myelitis. In Patient #9, the MRI showed the presence of a lesion in the posterior horn (C2-7). Similarly, Galassi et al. reported longitudinally extensive transverse myelitis (LETM) in a 40-year-old woman with amphiphysin antibody positivity (22). A study also demonstrated post-mortem histopathological evidence of the presence of CD8+ T cell-mediated immune responses in the spinal cord and dorsal root ganglia (17), confirming the involvement of the spinal cord.

Sensory neuronopathy has been found to be closely related to the presence of amphiphysin antibodies by previous studies (23). Our two patients, however, despite their complaints of paresthesia, did not meet the criteria of sensory neuronopathy (24). Besides, it had been considered that involvement of motor nerve or CSF inflammatory may indicate a paraneoplastic origin (25).

Amphiphysin is an intraneuronal protein that is targeted in 5-10% of PNS cases, and the presence of anti-amphiphysin antibodies has been listed as a significant risk factor for tumor occurrence (>80%) (23). Amphiphysin antibodies are widely known to be associated with both breast and small-cell lung cancers. Associations with other cancers have also been reported, including angiosarcoma and thyroid adenoma (**Supplementary Table 1**). Nevertheless, a much lower rate of cancer (2/10) was found in this study (19). No new neoplasms were diagnosed after discharge in our patient cohort. It is possible that insufficient follow-up may partially account for our low detection rate. Repeated screening every four or six months for two years is recommended (23). Accordingly, the necessity of cancer screening must be emphasized, and systemic tumor screening requires whole-body PET-CT as the first choice.

Amphiphysin antibodies are usually detected by immunoblotting, the gold standard for the identification of intracellular antibodies. Previous studies have reported the co-existence of amphiphysin antibodies with anti-neuronal nuclear antibody type 1 (ANNA1) and CRMP5 antibodies (17, 19). Here, we observed a co-occurrence of amphiphysin antibodies with CV2 and AMPAR antibodies. This overlap between amphiphysin antibodies and other subtypes of autoantibodies suggests that AE has both a complex etiology and mechanism. Furthermore, although not included in the present study, we have noticed amphiphysin antibody positivity in some AD (Alzheimer’s disease) and PD (Parkinson’s disease) patients. Although cognitive impairment may be a common manifestation, the progressive course of the disease suggests neurodegeneration rather than encephalitis. Thus, amphiphysin antibodies could be either causative agents or nonpathogenic bystanders caused by other CNS diseases. In terms of clinical diagnosis, the identification of this disease should thus be based on a thorough understanding of amphiphysin antibody pathology as well as clinical experience.

Patients with anti-amphiphysin encephalitis did not show distinctive features on either EEG or brain MRI. Involvement of the mesial temporal lobe was most common, which may mimic other subtypes of AE. Furthermore, involvement of the thalamus was found in one patient, consistent with the findings of a previous report (10). PET-CT revealed evidence of abnormal metabolism in several brain regions, although these were not specific. With the development of imaging analyses, more attention focusing on characteristic structural and functional imaging patterns is needed.

In our study, most patients received IVIG or corticosteroids along with ASMs, and their mRS scores reduced at follow-up. In animal experiments, animals exposed to purified amphiphysin antibodies showed symptom relief after plasmapheresis (26, 27). Clinically, a combination of immunotherapy and tumor treating is required (28). Besides, responses to high-dose benzodiazepine have also been noted (18). Follow-ups of the patients showed that the duration of symptoms varied under standard therapy. Although the seizure frequencies were observed to be reduced during follow-ups of 1 to 33 months, there was minimal improvement in cognitive impairment, sleep disturbances, or psychotic mood disorders. By tracking for 13 years, Taube et al. observed a similar outcome in a patient who was treated promptly with immunotherapy (29). As in other forms of AE, anti-amphiphysin encephalitis presenting with acute symptomatic seizures has a lower risk of subsequent unprovoked seizure development, which perhaps explains the disappearance of seizures in most patients.

There are some limitations in this study. Firstly, the sample size may have been too small. Secondly, immunoblotting is a semi-quantitative assay that does not show the exact titers of antibodies and may be subject to false-positive or false-negative results. In addition, in some cases, the absence of important examination findings such as electromyography and polysomnography hampered physician awareness of the disease and its treatment. In the future, the sample size should be expanded for more in-depth research.

## Conclusion

Amphiphysin-antibody-related encephalitis is a rare type of autoimmune encephalitis. The most common manifestation was limbic encephalitis and a lower association with cancer was observed compared with that reported in other PNS cohorts. Although seizure frequencies declined in response to immunotherapy, psychiatric and cognitive symptoms, as well as sleep disturbances, often remained. The awareness of the clinical features of amphiphysin antibody-related encephalitis provides valuable information for a better understanding of the disease and may help to facilitate its early diagnosis, treatment strategy, and prognosis prediction.

## Data availability statement

The original contributions presented in the study are included in the article/**Supplementary Material**. Further inquiries can be directed to the corresponding authos.

## Ethics statement

The studies involving human participants were reviewed and approved by KY2021-088-03. The patients/participants provided their written informed consent to participate in this study. Written informed consent was obtained from the individual(s) for the publication of any potentially identifiable images or data included in this article.

## Author contributions

QW and YS concepted, designed, and supervised the study. YS, XQ and DH acquired the data. YS and XQ analyzed and interpreted the data, provided statistical analysis, had full access to all of the data in the study, and are responsible for the integrity of the data and the accuracy of the data analysis. YS and XQ drafted the manuscript. ZZ, YZ and QW critically revised the manuscript for important intellectual content. All authors contributed to the article and approved the submitted version.

## References

[1] Graus F Titulaer MJ Balu R Benseler S Bien CG Cellucci T. A clinical approach to diagnosis of autoimmune encephalitis. Lancet Neurol (2016) 15:391–404. doi: 10.1016/s1474-4422(15)00401-9 26906964 PMC5066574

[2] Dubey D Pittock SJ Kelly CR Mckeon A Lopez-Chiriboga AS Lennon VA. Autoimmune encephalitis epidemiology and a comparison to infectious encephalitis. Ann Neurol (2018) 83:166–77. doi: 10.1002/ana.25131 PMC6011827 29293273

[3] Broadley J Seneviratne U Beech P Buzzard K Butzkueven H O'brien T. Prognosticating autoimmune encephalitis: A systematic review. J Autoimmun (2019) 96:24–34. doi: 10.1016/j.jaut.2018.10.014 30595145

[4] Dalmau J Tüzün E Wu HY Masjuan J Rossi JE Voloschin A. Paraneoplastic anti-N-methyl-D-aspartate receptor encephalitis associated with ovarian teratoma. Ann Neurol (2007) 61:25–36. doi: 10.1002/ana.21050 17262855 PMC2430743

[5] Pruss H Kirmse K. Pathogenic role of autoantibodies against inhibitory synapses. Brain Res (2018) 1701:146–52. doi: 10.1016/j.brainres.2018.09.009 30205110

[6] Evergren E Marcucci M Tomilin N Low P Slepnev V Andersson F. Amphiphysin is a component of clathrin coats formed during synaptic vesicle recycling at the lamprey giant synapse. Traffic. (2004) 5:514–28. doi: 10.1111/j.1398-9219.2004.00198.x 15180828

[7] McCracken L Zhang J Greene M Crivaro A Gonzalez J Kamoun M. Improving the antibody-based evaluation of autoimmune encephalitis. Neurol Neuroimmunol Neuroinflamm. (2017) 4:e404. doi: 10.1212/nxi.0000000000000404 29075658 PMC5639462

[8] Saiz A Dalmau J Butler MH Chen Q Delattre JY De Camilli P. Anti-amphiphysin I antibodies in patients with paraneoplastic neurological disorders associated with small cell lung carcinoma. J Neurol Neurosurg Psychiatry (1999) 66:214–7. doi: 10.1136/jnnp.66.2.214 PMC1736210 10071102

[9] Krishna VR Knievel K Ladha S Sivakumar K. Lower extremity predominant stiff-person syndrome and limbic encephalitis with amphiphysin antibodies in breast cancer. J Clin Neuromuscul Dis (2012) 14:72–4. doi: 10.1097/CND.0b013e31826f0d99 23172386

[10] Moon J Lee ST Shin JW Byun JI Lim JA Shin YW. Non-stiff anti-amphiphysin syndrome: Clinical manifestations and outcome after immunotherapy. J Neuroimmunol. (2014) 274:209–14. doi: 10.1016/j.jneuroim.2014.07.011 25087755

[11] Coppens T Van Den Bergh P Duprez TJ Jeanjean A De Ridder F Sindic CJ. Paraneoplastic rhombencephalitis and brachial plexopathy in two cases of amphiphysin auto-immunity. Eur Neurol (2006) 55:80–3. doi: 10.1159/000092307 16567945

[12] Chamard L Magnin E Berger E Hagenkötter B Rumbach L Bataillard M. Stiff leg syndrome and myelitis with anti-amphiphysin antibodies: A common physiopathology? Eur Neurol (2011) 66:253–5. doi: 10.1159/000331592 21986240

[13] Flanagan EP Mckeon A Lennon VA Kearns J Weinshenker BG Krecke KN. Paraneoplastic isolated myelopathy: clinical course and neuroimaging clues. Neurology. (2011) 76:2089–95. doi: 10.1212/WNL.0b013e31821f468f 21670438

[14] Perego L Previtali SC Nemni R Longhi R Carandente O Saibene A. Autoantibodies to amphiphysin I and amphiphysin II in a patient with sensory-motor neuropathy. Eur Neurol (2002) 47:196–200. doi: 10.1159/000057898 12037431

[15] Wesselingh R Butzkueven H Buzzard K Tarlinton D O'brien TJ Monif M. Innate immunity in the central nervous system: A missing piece of the autoimmune encephalitis puzzle? Front Immunol (2019) 10:2066. doi: 10.3389/fimmu.2019.02066 31552027 PMC6746826

[16] Shaw PJ. Stiff-man syndrome and its variants. Lancet. (1999) 353:86–7. doi: 10.1016/S0140-6736(05)76151-1 10023890

[17] Pittock SJ Lucchinetti CF Parisi JE Benarroch EE Mokri B Stephan CL. Amphiphysin autoimmunity: Paraneoplastic accompaniments. Ann Neurol (2005) 58:96–107. doi: 10.1002/ana.20529 15984030

[18] Murinson BB Guarnaccia JB. Stiff-person syndrome with amphiphysin antibodies: distinctive features of a rare disease. Neurology. (2008) 71:1955–8. doi: 10.1212/01.wnl.0000327342.58936.e0 PMC2676978 18971449

[19] Dubey D Jitprapaikulsan J Bi H Do Campo RV Mckeon A Pittock SJ. Amphiphysin-IgG autoimmune neuropathy: A recognizable clinicopathologic syndrome. Neurology. (2019) 93:e1873–80. doi: 10.1212/WNL.0000000000008472 31624089

[20] Xie YY Meng HM Zhang FX Maimaiti B Jiang T Yang Y. Involuntary movement in stiff-person syndrome with amphiphysin antibodies: A case report. Med (Baltimore). (2021) 100:e24312. doi: 10.1097/MD.0000000000024312 PMC7837982 33546061

[21] Martinez-Hernandez E Arino H Mckeon A Iizuka T Titulaer MJ Simabukuro MM. Clinical and immunologic investigations in patients with stiff-person spectrum disorder. JAMA Neurol (2016) 73:714–20. doi: 10.1001/jamaneurol.2016.0133 PMC5020136 27065452

[22] Galassi G Ariatti A Rovati R Genovese M Rivasi F. Longitudinally extensive transverse myelitis associated with amphiphysin autoimmunity and breast cancer: a paraneoplastic accompaniment. Acta Neurol Belg. (2016) 116:395–7. doi: 10.1007/s13760-015-0534-9 26358952

[23] Graus F Vogrig A Muniz-Castrillo S Antoine JG Desestret V Dubey D. Updated diagnostic criteria for paraneoplastic neurologic syndromes. Neurol Neuroimmunol Neuroinflamm. (2021) 8 (4):e1014. doi: 10.1212/NXI.0000000000001014 PMC8237398 34006622

[24] Camdessanche JP Jousserand G Ferraud K Vial C Petiot P Honnorat J. The pattern and diagnostic criteria of sensory neuronopathy: a case-control study. Brain. (2009) 132:1723–33. doi: 10.1093/brain/awp136 PMC2702838 19506068

[25] Camdessanche JP Jousserand G Franques J Pouget J Delmont E Creange A. A clinical pattern-based etiological diagnostic strategy for sensory neuronopathies: a French collaborative study. J Peripher Nerv Syst (2012) 17:331–40. doi: 10.1111/j.1529-8027.2012.00411.x 22971095

[26] Sommer C Weishaupt A Brinkhoff J Biko L Wessig C Gold R. Paraneoplastic stiff-person syndrome: Passive transfer to rats by means of IgG antibodies to amphiphysin. Lancet (2005) 365:1406–11. doi: 10.1016/s0140-6736(05)66376-3 15836889

[27] Baizabal-Carvallo JF. The neurological syndromes associated with glutamic acid decarboxylase antibodies. J Autoimmun (2019) 101:35–47. doi: 10.1016/j.jaut.2019.04.007 31000408

[28] Schmierer K Valdueza JM Bender A Decamilli P David C Solimena M. Atypical stiff-person syndrome with spinal MRI findings, amphiphysin autoantibodies, and immunosuppression. Neurology. (1998) 51:250–2. doi: 10.1212/wnl.51.1.250 9674811

[29] Taube J Witt JA Baumgartner T Helmstaedter C. All's well that ends well? long-term course of a patient with anti-amphiphysin associated limbic encephalitis. Epilepsy Behav Rep (2022) 18:100534. doi: 10.1016/j.ebr.2022.100534 35360257 PMC8960971

