# Supplementary material for: Anti-amphiphysin encephalitis: Expanding the clinical spectrum
Source: Front Immunol. 2023 Apr 5;14:1084883. doi: 10.3389/fimmu.2023.1084883 (PMC10113538; doi:10.3389/fimmu.2023.1084883)
Supplement: Supplementary file 1 [file Table_1.docx]

Supplementary material

# Supplementary Table 1

| **#of patients** | **Demographic information** | **Clinical characteristics** | **Tumor** | **Auxiliary examination** | **Lab test** | **Treatment** | **Prognosis and outcomes** |
| --- | --- | --- | --- | --- | --- | --- | --- |
| 1(xie et al., 2021) | N = 1  69y/ male | Stiff-person syndrome  Dyspepsia  Mandibular involuntary movement | Not found | **Brain and spinal MRI:** normal  **EEG:** no epileptiform activity  **EMG:** continuous motor activity in the biceps and deltoid muscles when the patient was relaxed | Anti-amphiphysin IgG in serum | Clonazepam (1mg/d) and baclofen (5mg, 3 times a day)  Corticosteroid iv (methylprednisolone 80 mg/d,7 days) followed by prednisone orally (60mg/d, reduced by 5mg weekly until discontinued)  **Maintenance therapy:** clonazepam (1mg/d) and baclofen (5mg, 3 times a day) | **Short-term outcomes:** muscle stiffness and dyspnea were obviously relieved  Involuntary movement of the mandible persisted throughout the treatment process  **Long-term outcomes (**in one year): muscle stiffness, dyspnea, and mandibular involuntary movement were still present |
| 2(mckeon, pittock, & lennon, 2009) | N = 11  female (n = 11, 100%)  median age:58y (range 39 –73) | Stiffness or rigidity (n = 10, 100%)  Spasms (n = 7, 70%)  Pain (n = 5, 50%) | Breast cancer (n = 10, 90%) | **EMG:** positive (n = 8, 100%): continuous motor unit activity or noted to be consistent with SPS | Anti-amphiphysin antibodies (+) | Steroid responsive (n = 4)  IVIG efficacy was not reported  “Dramatically better” following tumor excision and chemotherapy (n = 3), not respond to this intervention and subsequently died (n = 1), symptom-free with immunosuppression prior to breast cancer diagnosis and treatment (n = 1). | Good prognosis (n = 8)  Bad prognosis (n = 1) |
| 3(krishna, knievel, ladha, & sivakumar, 2012) | N = 1  54y/ female | Panic attacks  Visual hallucinations  Painful extensor spasms of the lower extremities  Partial-onset seizures; generalized tonic–clonic seizure | Breast cancer (ductal breast carcinoma) | **MRI:** hyperintensity in the left temporal lobe with enhancement.  **Chest CT:** enlarged (2.4 × 1 cm) right axillary lymph node.  The lymph node was biopsied and showed metastatic ductal breast carcinoma | Amphiphysin-abs were elevated in the serum | **Tumor therapy:** chemotherapy (cyclophosphamide and epirubicin, followed by docetaxel) and conservative breast irradiation  **Maintenance therapy:** low doses of baclofen and diazepam  Levetiracetam was successfully tapered several weeks after her treatment | Dramatic improvement in her stiffness and spasms and her recovery of the tendon reflexes  No further episodic affective or sensory symptoms  **MRI:** brain MRI at 1-year follow-up showed partial resolution of the temporal lobe abnormality and enhancement |
| 4(moon et al., 2014) | N = 20  male (n = 12, 60%)  mean age (57.6 ± 17.2 y) | Limbic encephalitis (n = 10)  Dysautonomia (n = 9)  Cerebellar dysfunction (n = 6)  Peripheral neuropathy (n = 4)  Myelitis (n = 1) | **Cancer (n = 7):**  Non-small cell lung cancer (n = 1)  Small cell lung cancer (n = 1)  Ovarian cancer (n = 1)  Cervical cancer (n = 1)  Esophageal cancer (n = 1)  Gastric cancer (n = 2) | **Brain MRI:** Parenchymal T2 high signal intensity (HSI) (n = 3), including periventricular white matter, basal ganglia and thalami and insula, frontal, tempo-occipital lobe leptomeningeal enhancement (n = 2)  T2 HSI lesion in upper cervical spinal cord (n = 1) | Additional autoantibodies (Hu, Yo, Ri, and NMDA antibodies) were detected in four patients | **Immunotherapy:** IVIG (n = 12), corticosteroids (n = 9), tacrolimus (n = 4), rituximab (n = 3), cyclophosphamide (n = 1), tocilizumab (n = 1), mycophenolate mofetil (n = 1) | Good prognosis (n = 11)  In three patients, symptoms improved after treatment with rituximab but not after treatment with IVIG or corticosteroids  After treatment, mRS decreased in eight patients, 11 were able to walk unassisted  Bad prognosis (n = 1)  He died due to an unrelated complication |
| 5(chou et al., 2013; lin, chou, lin, & wang, 2011) | N = 3  median age 11y (range 9-13)  female (n = 1, 33%) | Limbic encephalitis  Encephalopathy  Memory impairment  Refractory temporal seizures (n = 3, 100%) | Not found (0/3) | **EEG:** multifocal epileptiform discharges (1/1)  **MRI:** FLAIR signal abnormalities in mediotemporal area or T2 hyperintensity in the temporal lobes (3/3) | **CSF:** anti-amphiphysin antibodies were found in serum (3/3) | **Immunotherapy:** intravenous methylprednisolone pulse therapy (30 mg/kg/dose once daily for 3 days) (n = 2)  Methylprednisolone at a dose of 20 mg/kg/day was given intravenously for 3 days, followed by 2 mg/kg/day of oral prednisolone (n = 1) | Relapse: 0/3  One left mental retardation  One left hallucination  One was discharged after 6 weeks of admission with minimal cognitive difficultly and motor disability |
| 6(chamard et al., 2011) | N = 1  65y/ female | Stiff person syndrome  Progressive encephalomyelitis with rigidity  Jerking stiff man syndrome  Stiff leg syndrome | Breast cancer (infiltrating canal carcinoma) (carcinoma was pathologically confirmed) | **Cerebral and medullary MRI:** normal  **PET-CT:** an anomaly on the right breast | Anti-amphiphysin antibodies were found in serum and CSF | **Acute management**: intravenous corticotherapy (1 g/day for 5 days), plus diazepam (5 mg 3 times per day) and baclofene (10 mg 3 times per day)  Cancer was treated by surgery, radiotherapy, and chemotherapy  **Maintenance therapy:** tizanidine | **Short-time outcomes:**  Spasms receded after 3 days, and the patient was able to walk again  Symptoms improved with the introduction of chemotherapy. After the fifth round, Stiff Leg Syndrome worsened with no associated neurological deficit  **Long-tome outcomes (**6 months after disease onset**):**  Unexpected transverse myelitis with a t2 hypersignal from level th6 to th10  One year after disease onset: the patient could walk with two sticks and no longer presented with spasms; however, she still suffered from stiff lower limbs  With no pyramidal syndrome; no further medullar abnormalities were found in the medullary MRI carried out 3 months later |
| 7(coppens et al., 2006) | N = 2  *Case 1#* 59y/ male  *Case 2#* 77y/ female | *Case 1#*  Dysarthria  Right limb ataxia  Right trigeminal paresthesia  Progressive cerebellar static ataxia  Right facial and common oculomotor nerve palsies  *Case 2#*  Left brachial neuropathy  Progressive sensory ataxia | *Case 1#*  Small cell lung carcinoma  *Case 2#*  A history of right breast cancer treated by surgery 16 years earlier | *Case1#*  **MRI:** normal  **PET-CT:** showed right cerebellar and occipital hypoperfusion  *Case2#*  **MRI:** brain and spinal MRIs were normal; unilateral hyperintense signal in the left brachial plexus  **PET-CT**: a single, enlarged, left axillary lymph node was detected  **EMG:** sensorimotor neuropathy with involvement of the posterior tracts of the spinal cord. | *Case 1#*  Anti-amphiphysin antibodies were found in the serum  *Case 2#*  Anti-amphiphysin antibodies were found in the serum | *Case 1#*  Chemotherapy  *Case 2#*  Radiotherapy and corticosteroid therapy (prednisone 48 mg/day for 6 weeks) | *Case 1#*  The patient developed brain metastases despite chemotherapy and died  *Case 2#*  Sensory deficit and gait disturbance worsened after treatment  The patient died from sepsis under recurrent antibiotic treatment for septic arthritis of the right shoulder |
| 7(vacaras, cucu, radu, & muresanu, 2020) | N = 1  68y/ female | Stiff person syndrome (bilateral lower-extremity spasm and severe pain)  Urine retention | Breast cancer (invasive no special type carcinoma) | **Cervical MRI:** degenerative modifications and discopathy with no evident nerve root conflicts  **Cerebral MRI:** a left bulbar ischemic lesion and a calcified superior frontal meningioma  **EMG:** no significant changes | Anti-amphiphysin antibodies were positive in the serum | High doses of benzodiazepines (diazepam and clonazepam), baclofen, levetiracetam and gabapentin  **Symptomatic treatment for pain relief** (botulinum toxin intramuscular locally, fentanyl patch 25 µg/72h and cannabidiol oil CBD 1,350 mg/100 ml) | During hospitalization, the physical and mental state of the patient degraded, then the patient showed respiratory failure and died of cardiac arrest |
| 8(neshige et al., 2014) | N = 1  5y/ female | Subacute sensory neuropathy with a longitudinally extensive spinal cord lesion of dorsal column and a dorsal root ganglion (DRG) lesion | Breast cancer (progesterone and estrogen receptor positive) (carcinoma was pathologically confirmed) | **Cervical MRI**: longitudinally extensive spinal cord lesion of the dorsal column at levels C1-T11  **Enhanced CT**: enlarged and fluorodeoxyglucose accumulation in parasternal lymph node  **PET-CT:** high FDG-uptake lesion in the left parasternal lymph node, but no lesion was detected in the breast | Anti-AQP4 (-)  Anti-amphiphysin (+) | **Cancer treatment:** tamoxifen and radiation therapy (up to now) | NA |
| 9(dubey et al., 2019) | N = 21  mean age: 45y (range47-79)  female (n = 19, 91%) | Pain (n = 16, 76%)  Stiff person spectrum disorder (n = 9, 45%)  Myelopathy (n = 6, 30%)  Cerebellar ataxia (n = 4, 19%)  Polyradiculoneuropathy (n = 13, 62%)  Sensory neuronopathy (n = 7, 35%)  Autonomic dysfunction (n = 1, 5%) | Breast cancer (n = 12, 63%)  Small cell lung cancer (n = 4, 22%) | **EMG (n = 19):**  Extremity motor and sensory nerve conduction amplitudes were reduced (n = 14)  Needle EMG showed spontaneous insertional activity and motor neurogenic changes (n = 9) | CSF inflammatory (7/13, 54%) | **Immunotherapy** (n = 15)  Iv methylprednisolone (IVMP) (1g daily for 5 days, N = 8), IV immunoglobulin (n = 8), plasmapheresis (n = 4), oral prednisone (n =7), cyclophosphamide (n = 5), rituximab (n = 1), and mycophenolate mofetil (n = 1) | Improvement (7/16, 44%)  Death within 5 years (6/21, 39%) |
| 10(hirunagi et al., 2016) | N = 1  61y/ female | Cerebellar ataxia | Follicular thyroid adenoma | **MRI:** cerebellar atrophy  **Contrast-enhanced CT:** left thyroid mass compressing the trachea  **EEG, EMG, spinal MRI, 、PET-CT:** normal | Positive anti-amphiphysin antibodies in serum | **Tumor therapy:** left lobe of the thyroid gland was resected  **Immunotherapy:** steroid pulse (methylprednisolone 1g/d for 3 days), IVIG (400mg/d for 5 days) and cyclophosphamide 750mg/m^2^ for 1 day | After treatment, cerebellar ataxia symptoms did not progress, but remaining lower limbs weakness. |
| 11(wagner-altendorf, wandinger, frydrychowicz, merseburger, & munte, 2019) | N = 1  72y/ male | Cognitive decline (Dysexecutive syndrome)  Mild anemia | Aortic angiosarcoma (metastases to kidney, muscle and bones) | **PET-CT:** tumor resulted in hypermetabolic foci in the descending thoracic aorta, the left kidney, the right iliac bone and the right paraspinal musculature suspicious of a metastasized renal cell carcinoma | **CSF:** highly positive anti-amphiphysin antibodies | High-dose glucocorticoids (methylprednisolone 1000mg/day intravenous for 3 days) followed by immunoglobulins (30g/day intravenous for 5 days)  Cytoreductive laparoscopic nephrectomy and a chemotherapy with weekly application of docetaxel (30mg/m²) | Clinical neurological symptoms had worsened  The patient died at home about 4 months later |
| 12(galassi, ariatti, rovati, genovese, & rivasi, 2016) | N = 1  40y/ female | Longitudinally extensive transverse myelitis (subacute onset of distal paresthesia and extremity weakness) | Breast cancer (invasive ductal carcinoma C-Erb B2 negative, positive for estrogen (95%) and progesterone receptors (15%) with low cytoproliferation activity (2%) | **Electrophysiology:** motor slowing along tibial, peroneal nerves within 35 and 39 m/sec, normal sensory velocity throughout, and increased latencies of somatosensory evoked responses in the limbs  **Contrast-enhanced MRI:** T2-weighted images showed symmetric linear hyperintensity  **Brain MRI and ophthalmological assessments:** normal  **CT, mammogram, chest MRI** revealed a lymphadenopathy satellite of focal enhancing lesion in the left breast. | Positive amphiphysin autoantibodies in serum and CSF | **Immunotherapy:** intravenous immunoglobulin (IVIG, 0.4 g/kg/bw daily for 5 days) followed by methylprednisolone (1g daily for 3 days) was given from day 10th  **Cancer treatment** included 4 courses of chemotherapy (5-fluorouracil 600 mg/kg, epidoxorubicin 90 mg/kg, and cyclophosphamide 600 mg/Kg). Radical surgery was performed 6 months after neurological onset. | 24 months after onset, neuroradiological abnormalities persisted associated with mild cord atrophy. The patient remained wheelchair bound. |
| 13(vinjam, shanmugarajah, & ford, 2016) | N = 1  47y/ female | Ophthalmoplegia  Stiff person syndrome  Respiratory failure and dysphagia | Breast cancer (grade 2 invasive ductal adenocarcinoma of left breast) | **MRI (brain and spine with contrast):** normal | Amphiphysin autoantibodies in serum | Non-invasive ventilation and nasogastric feeding  **Immunotherapy:** 5-day course of intravenous immunoglobulins, followed by five cycles of plasma exchange which resulted in only partial resolution of the stiffness  **Cancer therapy:** commenced on six cycles of adjuvant chemotherapy with epirubicin and cyclophosphamide | Weaned from ventilator support and the dysphagia, painful stiffness and rigidity resolved gradually. The restricted eye movements significantly improved. |

chamard, l., magnin, e., berger, e., hagenkotter, b., rumbach, l., & bataillard, m. (2011). Stiff leg syndrome and myelitis with anti-amphiphysin antibodies: a common physiopathology? *Eur neurol, 66*(5), 253-255. doi:10.1159/000331592

Chou, i. J., wang, h. S., lin, j. J., kuo, c. F., lin, k. L., chou, m. L., . . . Group, c. S. (2013). Limbic encephalitis in taiwanese children and adolescence: a single center study. *Pediatr neonatol, 54*(4), 246-253. doi:10.1016/j.pedneo.2013.01.016

Coppens, t., van den bergh, p., duprez, t. J., jeanjean, a., de ridder, f., & sindic, c. J. (2006). Paraneoplastic rhombencephalitis and brachial plexopathy in two cases of amphiphysin auto-immunity. *Eur neurol, 55*(2), 80-83. doi:10.1159/000092307

Dubey, d., jitprapaikulsan, j., bi, h., do campo, r. V., mckeon, a., pittock, s. J., . . . Klein, c. J. (2019). Amphiphysin-IgGautoimmune neuropathy: a recognizable clinicopathologic syndrome. *Neurology, 93*(20), e1873-e1880. doi:10.1212/wnl.0000000000008472

Galassi, g., ariatti, a., rovati, r., genovese, m., & rivasi, f. (2016). Longitudinally extensive transverse myelitis associated with amphiphysin autoimmunity and breast cancer: a paraneoplastic accompaniment. *Acta neurol belg, 116*(3), 395-397. doi:10.1007/s13760-015-0534-9

Hirunagi, t., sato, k., fujino, m., tanaka, k., goto, y., & mano, k. (2016). Subacute cerebellar ataxia with amphiphysin antibody developing in a patient with follicular thyroid adenoma: a case report. *Rinsho shinkeigaku, 56*(11), 769-772. doi:10.5692/clinicalneurol.cn-000939

Krishna, v. R., knievel, k., ladha, s., & sivakumar, k. (2012). Lower extremity predominant stiff-person syndrome and limbic encephalitis with amphiphysin antibodies in breast cancer. *J clin neuromuscul dis, 14*(2), 72-74. doi:10.1097/cnd.0b013e31826f0d99

Lin, j. J., chou, i. J., lin, k. L., & wang, h. S. (2011). Childhood refractory focal epilepsy following acute febrile encephalopathy with anti-amphiphysin antibody. *Eur j neurol, 18*(6), e70. doi:10.1111/j.1468-1331.2010.03342.x

Mckeon, a., pittock, s. J., & lennon, v. A. (2009). Stiff-person syndrome with amphiphysin antibodies: distinctive features of a rare disease. *Neurology, 73*(24), 2132; author reply 2133. doi:10.1212/wnl.0b013e3181bd6a72

Moon, j., lee, s. T., shin, j. W., byun, j. I., lim, j. A., shin, y. W., . . . CHu, k. (2014). Non-stiff anti-amphiphysin syndrome: clinical manifestations and outcome after immunotherapy. *J neuroimmunol, 274*(1-2), 209-214. doi:10.1016/j.jneuroim.2014.07.011

Neshige, s., hara, n., takeshima, s., iwaki, h., shimoe, y., takamatsu, k., & kuriyama, m. (2014). [anti-amphiphysin antibody-positive paraneoplastic neurological syndrome with a longitudinally extensive spinal cord lesion of the dorsal column]. *Rinsho shinkeigaku, 54*(7), 572-576. doi:10.5692/clinicalneurol.54.572

Vacaras, v., cucu, e. E., radu, r., & muresanu, d. F. (2020). Paraneoplastic stiff person syndrome in early-stage breast cancer with positive anti-amphiphysin antibodies. *Case rep neurol, 12*(3), 339-347. doi:10.1159/000508942

Vinjam, m. R., shanmugarajah, p., & ford, h. L. (2016). Ophthalmoplegia heralding the onset of anti-amphiphysin related paraneoplastic stiff person syndrome. *J neurol, 263*(5), 1017-1018. doi:10.1007/s00415-016-8078-3

Wagner-altendorf, t. A., wandinger, k. P., frydrychowicz, a., merseburger, a. S., & munte, t. F. (2019). Anti-amphiphysin-associated limbic encephalitis in a 72-year-old patient with aortic angiosarcoma. *Bmj case rep, 12*(3). Doi:10.1136/bcr-2018-226798

Xie, y. Y., meng, h. M., zhang, f. X., maimaiti, b., jiang, t., & yang, y. (2021). Involuntary movement in stiff-person syndrome with amphiphysin antibodies: a case report. *Medicine (baltimore), 100*(3), e24312. doi:10.1097/md.0000000000024312

# Supplementary Table 2

| Patient | Anti-amphiphysin antibodies | | MRI | | EEG | | PET-CT | |
| --- | --- | --- | --- | --- | --- | --- | --- | --- |
|  | First detection | Reexamination | First detection | Reexamination | First detection | Reexamination | First detection | Reexamination |
| 1 | +++ (serum+CSF) | +++ (serum) (3m) | Increased T2 signal in right MTL | No obvious changes (3m) | NA | NA | Right MTL hypermetabolism | NA |
| 2 | + (serum) | NA | Normal | NA | NA | NA | Right parietal lobe, bilateral frontal lobe hypometabolism, bilateral BG hypermetabolism | NA |
| 3 | + (serum) | NA | Increased T2 signal in left MTL | Reduction of volume in lesion (15m) | Sharp waves in left MTL | NA | Left MTL hypometabolism | NA |
| 4 | + (serum) | - (serum) (1m) | DWI showed diffusion restriction in bilateral thalamus with blurred structure | No obvious changes (13m) | NA | NA | Bilateral central cortex, left fronto-parietal para-sagittal, BG, right thalamus hypermetabolism | NA |
| 5 | + (serum) | - (serum) (5m) | Decreased volume of left hippocampus | No obvious changes (5m) | Sharp waves in right frontal lobe and anterior-middle temporal cortex | Slow waves in right MTL (10m) | Right MTL hypometabolism | NA |
| 6 | + (serum) | NA | Increased T2 signal in the left MTL | NA | Normal | NA | Left MTL hypometabolism | NA |
| 7 | + (serum) | - (serum) (8m) | Normal | NA | Continuous sharp waves in the left central-parietal area | NA | Left MTL hypermetabolism | NA |
| 8 | + (serum) | NA | Normal | NA | NA | NA | NA | NA |
| 9 | ++ (serum) | + (serum) (12m) | Increased T2 signal and enhancement in posterior horn of the spinal cord (C2-7) | No obvious changes (15m) | NA | NA | Spinal cord hypermetabolism | NA |
| 10 | + (serum) | NA | Increased T2 signal in bilateral MTL | NA | Normal | NA | NA | NA |

*Abbreviation: BG, basal ganglia; m, month; MTL, medial temporal lobe; NA, not applicable.*
